# m^7^G-Related DNA Damage Repair Genes are Potential Biomarkers for Predicting Prognosis and Immunotherapy Effectiveness in Colon Cancer Patients

**DOI:** 10.3389/fgene.2022.918159

**Published:** 2022-06-09

**Authors:** Shuran Chen, Rui Dong, Yan Li, Ni Zheng, Guisen Peng, Fei Lu, Quanwei Qiu, Hexin Wen, Yitong Wang, Huazhang Wu, Mulin Liu

**Affiliations:** ^1^ Department of Gastrointestinal Surgery, First Affiliated Hospital of Bengbu Medical College, Bengbu, China; ^2^ Department of Gynecologic Oncology, First Affiliated Hospital of Bengbu Medical College, Bengbu, China; ^3^ School of Life Science, Anhui Province Key Laboratory of Translational Cancer Research, Bengbu Medical College, Bengbu, China

**Keywords:** colon cancer, prognostic model, m 7 G, DNA damage repair, tumour immunity

## Abstract

**Objective:** m^7^G is a post-transcriptional modification modality, however, limited research has been conducted on its role in colon cancer. DNA damage repair (DDR) is an important factor that contributes to colon cancer development, growth and chemoresistance. This study aimed to explore whether m^7^G-related DNA damage repair genes may be used as biomarkers to predict the prognosis of colon cancer patients.

**Methods:** We use non-negative matrix factorization (NMF) to type CRC patients into. Risk models were constructed using different expression genes in two clusters. We assessed the reliability of risk models with DCA curves, and a Nomogram. Meanwhile, The receiver operating characteristic and C-index curves were used to compare the predictive significance of the constructed risk models with other studies. In additional, we examined the significance of risk models on patients’ immunity microenvironment and response to immune therapy. Finally, we used a series of cellular experiments to validate the effect of model genes on the malignant progression of CRC cells.

**Results:** Twenty-eight m^7^G genes were obtained from the GSEA database. Multivariate Cox and LASSO Cox regression analysis was performed and eleven m^7^G-related DDR genes were identified for constructing the risk model. Survival and stage of CRC patients were worser in the high-risk group than in the low-risk group for both the training and test sets. Additionally, the different immune microenvironment status of patients in the high- and low-risk groups, suggesting that patients in the low-risk group may be more sensitive to immunotherapy, particularly immune checkpoint inhibitors. Finally, we found that depletion of ATP2A1, one of the risk genes in our model, influence the biologic behaviour of CRC cells significantly.

**Conclusion:** The m^7^G-related DDR genes can be used as important markers for predicting patient prognosis and immunotherapy response. Our data suggest that ATP2A1 may promote the proliferation of colon cancer cells. These findings may provide new therapeutic targets for the treatment of colon cancer.

## Introduction

Colorectal cancer ranks third in terms of incidence and second in terms of mortality among neoplastic diseases (Sung et al., 2021). Worse still, colorectal cancer tends to be more prevalent in younger people (Lieu et al., 2019). With the advancement of molecular research on colorectal cancer, numerous serum tumour markers, and molecular screening techniques have significantly increased the detection rate of early-stage tumours (Russo et al., 2019; Miao et al., 2020). Patients with early-stage colorectal cancer have a significantly better response to treatment and prognosis as compared to those with advanced colorectal cancer (Andrew et al., 2018). Therefore, it is important to develop new diagnostic markers for the management of colorectal cancer.

Epigenetic dysregulation is strongly associated with the occurrence of a variety of diseases, particularly cancer (Nacev et al., 2020; Galassi et al., 2021). Almost all gastrointestinal tumours are caused by epigenetic dysregulation, but these alterations can be used to predict cancer risk, prognosis, and response to therapy (Grady et al., 2021). m^7^G methylation is a conserved modification found in both eukaryotes and bacteria (Jühling et al., 2009). Previous studies established that m^7^G occurs most frequently on tRNA and contributes to the stability of tRNA (Tomikawa, 2018). Recently, it was discovered that m^7^G has important modifying effects on other types of RNA (Lin et al., 2018; Dai et al., 2021; Ying et al., 2021). The development of colon cancer is closely related to defective DNA repair, chromosome instability, microsatellite instability, and alterations in the serrated pathway and DNA methylation. All of these features point DNA damage repair to the core to overcome the colorectal cancer. However, the effect of m^7^G-related DDR genes on the prognosis of colon cancer patients has not been previously reported.

In this study, we analysed the transcriptomic and clinical data from TCGA database of colon cancer patients and constructed a prognostic model for m^7^G-related DNA damage repair genes. The model was then validated with GEO datasets. Finally, we downgraded the expression of one of the ATP2A1 one of the model gene to explore its role in colon cancer. This model may provide a new reference for doctors to assess the prognosis of colon cancer patients. Meanwhile, targeting of ATP2A1 may become a potential therapeutic strategy for colon cancer.

## Material Methods

### Data Acquisition and Processing

The m^7^G and DNA damage repair genes data was obtained by a combination of the literature (Tomikawa, 2018) and the GSEA database (http://www.gsea-msigdb.org/gsea/index.jsp). Colon cancer patients’ transcriptomic, clinical, and mutation data were obtained from the TCGA database (https://portal.gdc.cancer.gov/)、GSE17536 and GSE39582. Batch effect in TCGA and GEO were removed by “limma” package. Only survival time for CRC patients is ≥ 30 days were retained, and 1,160 patients were finally enrolled in this study (Table 1).

**TABLE 1:** Information on patients included in this study.

	TCGA (n = 417)	GSE39582 (n = 566)	GSE17536 (n = 177)
**Gender**			
Female	193	256	81
Male	224	310	96
**OS**			
Alive	329	371	104
Dead	88	195	73
**T Stage**			
T1	9	11	
T2	74	45	
T3	284	367	
T4	49	119	
Unknow	1	24	
**N Stage**			
N0	247	302	
N1	98	134	
N2	72	98	
N3		6	
N4		26	
Unknow			
**M Stage**			
M0	311	482	
M1	57	61	
Unknow	49	23	
**Stage**			
I	72		24
II	160		57
III	117		57
IV	57		39
unknow	11		
**Age**			
<65	161		78
≥65	256		99

### NMF Consensus Clustering

David (https://david.ncifcrf.gov/) performed Gene Ontology (GO) and Kyoto Encyclopaedia of Genes and Genomes (KEGG) enrichment analysis of the m^7^G gene (Huang et al., 2009). The m^7^G-associated DNA damage repair gene was identified using the ‘psych’ package with R > 0.1 and adjusted *p*-value < 0.05. The DDR Subtypes were identified using the ‘NMF’ package in R software. The following criteria should be followed for a good clustering effect: 1. the cumulative distribution function curve increases smoothly; 2. the sample size of each group should not be too small, and after clustering, intra-group correlation is high and inter-group correlation is low. The “limma” package was used to screen for typing differences using a filter of |log2fold change (FC)| >0.1 and adjusted *p*-value <0.05. The “GSVA” package of the R software explored the different functional regions of the genes that differed between typing. ssGSEA was used to explore the association between typing and immune cell infiltration.

### Construction and Validation of a Prognostic Model

The 417 patients from TCGA were used as the train group for constructing the prognostic model and the 743 patients from the GEO database were used as the test group for the validation of the prognostic model. The patients were divided into two groups according to the median risk score, and the correlation heatmap and risk heatmap were drawn using the “Psych” and “pheatmap” packages. Principal component analysis (PCA) was performed to determine the validity of the risk model. Kaplan–Meier survival plots, receiver operating characteristic (ROC), C-index and DCA curves, visualized by R package“survival”, “timeROC”,“ggDCA” and “survminer”, were used to evaluate prognostic value of risk model in colon cancer. The constructed model in this study was also compared with known models in predicting the prognosis of colon patients (Huang et al., 2018; Li et al., 2021; Liang et al., 2021). Univariate and multifactor analyses were performed to determine the independent prognostic value of the risk model. Nomogram plots for the TCGA and GEO datasets were plotted separately using the ‘rms’ package of R software, and calibration curves were used to assess the accuracy of the column plots.

### Association Between the Risk Model and the Immune Microenvironment

“CIBERSORT” and “MCPcounter” package were used to analysed the different immune microenvironment between high- and low-risk group. The samples were filtered based on *p* < 0.05, and 1,000 simulations were performed to improve data accuracy. Heat and scatter plots of risk scores versus immune cell content were created using “ggplot2” and “tidyverse” packages. The difference in immune cell content between the high- and low-risk groups was determined using single-sample gene set enrichment analysis (ssGSEA).

### Associations Between Risk Models and Mutation, and Drug Sensitivity

To investigate the relationship between the risk model and tumour mutation, the “maftools” package was used to obtain the mutation status of patients in both high and low-risk groups. The “pRRophetic” package was used to predict the sensitivity of the risk model to clinical drug treatment. Colon cancer patient’s dates were obtained from the TIDE (http://tide.dfci.harvard.edu/).The relationship between risk scores and patient prognosis and immunotherapy efficacy was analysed using the pearson correlation test.

### The Expression and Biological Function of ATP2A1 in Colon Cancer

Western blot and Immunohistochemistry were used to detect the different expression of ATP2A1 in tumour and adjacent normal tissue. RKO、HCT116、SW620 and NCM460 were cultured in DMEM with 10% fatal bovine serum. Two small inferring RNAs (siRNAs) were employed to knock down ATP2A1 (siRNA#1, 5′-GGU​GGU​UCC​UGU​ACG​GUG​ATT-3′; siRNA#2, 5′-GAA​UGU​GUU​CAA​CAC​GGA​UTT-3′). CCK8 and EdU assays were used to test proliferation in control and ATP2A1 knockdown cells.

### Data Analysis

All statistical analyses were performed using R version 4.1.0. Statistical results were considered significant at *p* < 0.05.

## Results

### Functions of m^7^G Genes in Colon Cancer

**FIGURE 1:**
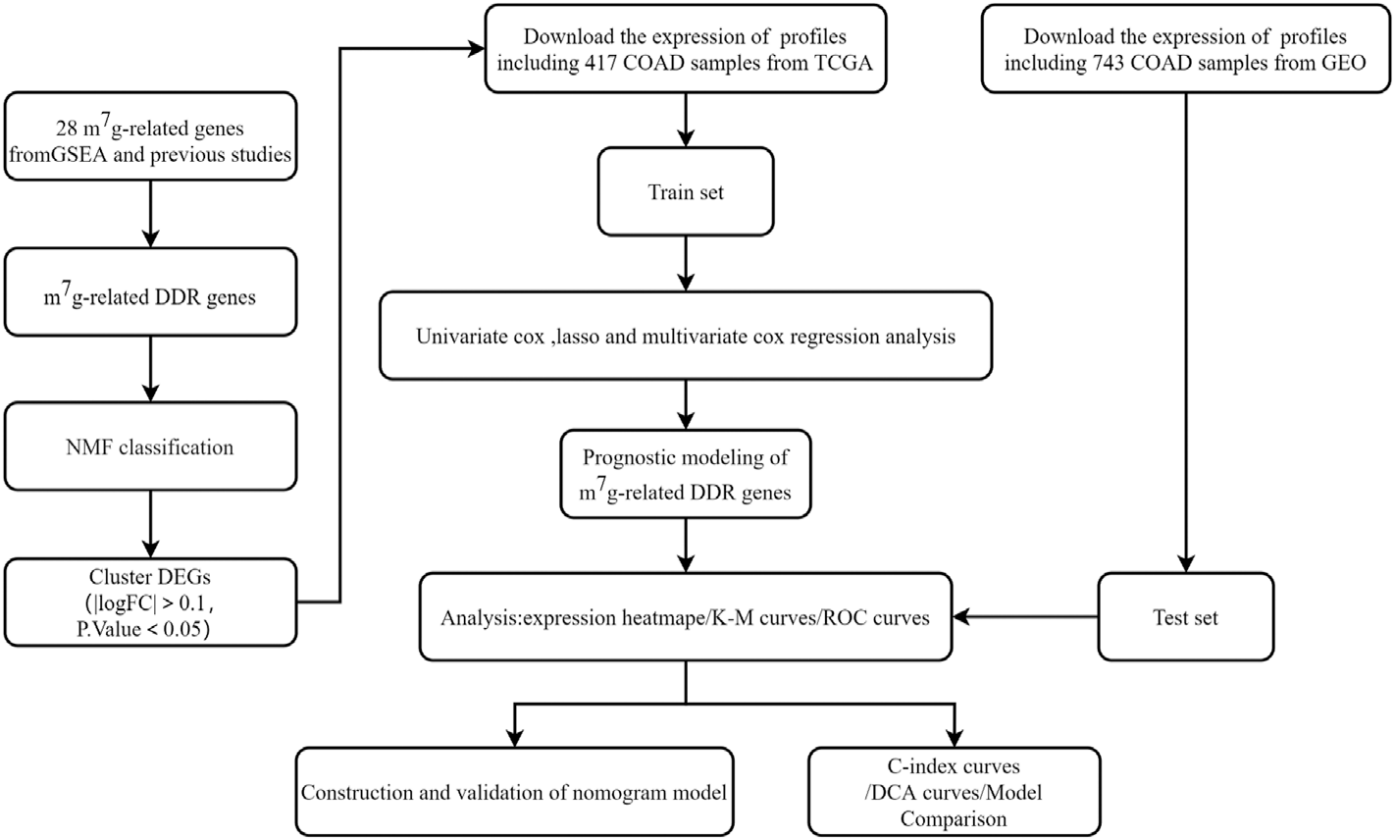
The flowchart showing the design to establish and validate the prognostic signature in the study.

**FIGURE 2:**
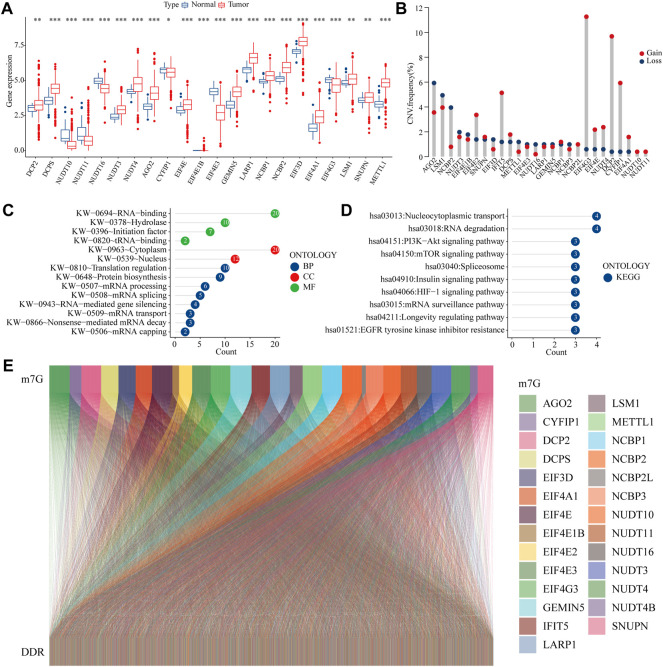
m^7^G related DDR gene **(A) **Expression of m^7^G-related genes in COAD and normal tissues **(B) **Mutation frequency of m^7^G-related genes **(C–D)** GO and KEGG analysis of m^7^G-related genes **(E)** Co-expression relationship between the m^7^G gene and DDR gene.

We first present a technical roadmap for building a risk model (Figure 1). Next, we investigate the expression of 28 m^7^G genes. The majority of m^7^G genes were significantly differentially expressed in cancer and normal tissues (Figure 2A). Additionally, most genes have varying degrees of mutation (Figure 2B). The GO and KEGG enrichment demonstrated that m^7^G genes are involved in both mRNA modification and resistance to tyrosine kinase inhibitors (Figures 2C,D). Resistance to tyrosine kinase inhibitors is relevant to methylation, meanwhile methylation of DDR genes is an important triggers of colon cancer. Therefore, we obtained DNA damage repair genes from GSEA official website and screened m^7^G-related DDR gene with correlation coefficient >0.1 (Figure 2E). In summary, we found that m^7^G may participate in regulate DNA repair in colon cancer.

### Distribution of m^7^G Related DDR Genes Subtypes Using NMF Consensus Clustering

**FIGURE 3:**
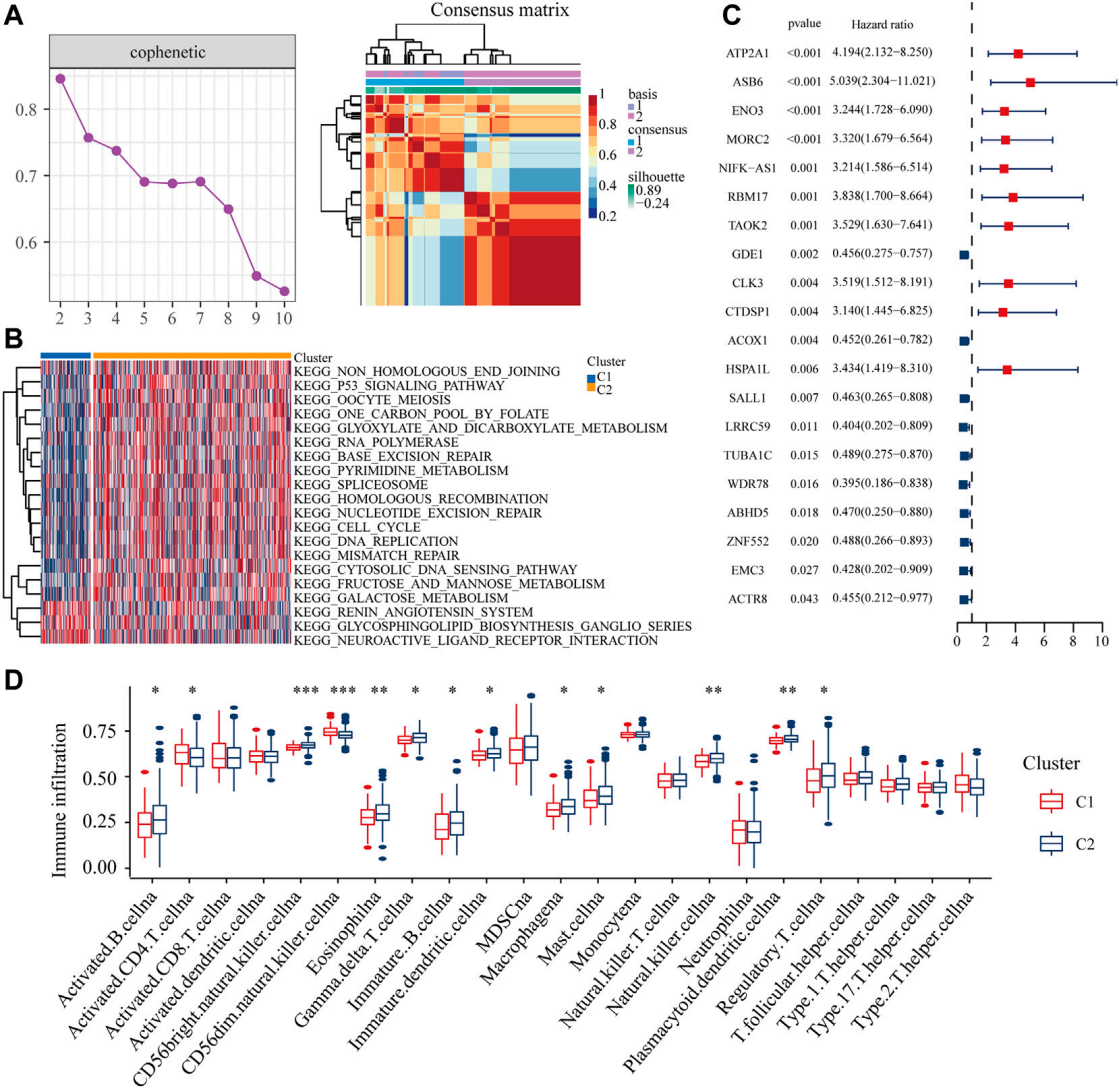
Distribution of m^7^G related DDR gene **(A)** NMF clustering based on m^7^G related DDR gene decomposes the samples in TCGA cohorts. **(B)** The GSVA analysis of two clusters. **(C)** The independent prognostic analysis of the DEGs from two clusters. **(D)** The ssGSEA analysis of two clusters.

According to cophenetic coefficients, we decided k = 2 as the best cluster number (Figure 3A: Supplementary Figure S1). When k = 2, including 84 cases in C1 subtype and 334 cases in C2 subtype (Supplementary Table S1). GSVA enrichment analysis shows that subtype C2 is significantly enriched in DNA damage repair related pathways such as mismatch repair, DNA repair and cell cycle (Figure 3B, Supplementary Table S2). In addition most of the differential expression genes have an independent prognostic effect on colon cancer patients (Supplementary Table S3). DNA damage repair is closely linked to the immune microenvironment of tumours. ssGSEA showed that the majority of immune cells had increased infiltration in subtype B (Figure 3D). Taken together, the m^7^G related DDR genes may regulate the immune microenvironment in colon cancer patients.

### Construction of the Risk Model

The TCGA dataset was used as the training set to construct the risk model, and the GSE17536 and GSE39582 datasets were used as the validation set to verify the value of the model (Supplementary Figure S2A, B). LASSO regression analysis and multivariate Cox analysis were performed on genes that differed between DDR subtypes to identify the best candidate genes. Multivariate Cox regression identified ten independent prognostic genes used for the construction of the risk models.

Risk Score=(0.363 * the expression of ATP2A1)+(0.215* the expression of HEYL)+(0.265* the expression of ASB6)+(0.298 * the expression of ZEB1. AS1)+(0.081 * the expression of NOL3)+(0.212* the expression of TRPM5)+(0.013 * the expression of HOXC6)+(0.0081 * the expression of DPP7)+(0.021 * the expression of GPRC5B)+(0.005 * the expression of PCDHB2).

**FIGURE 4:**
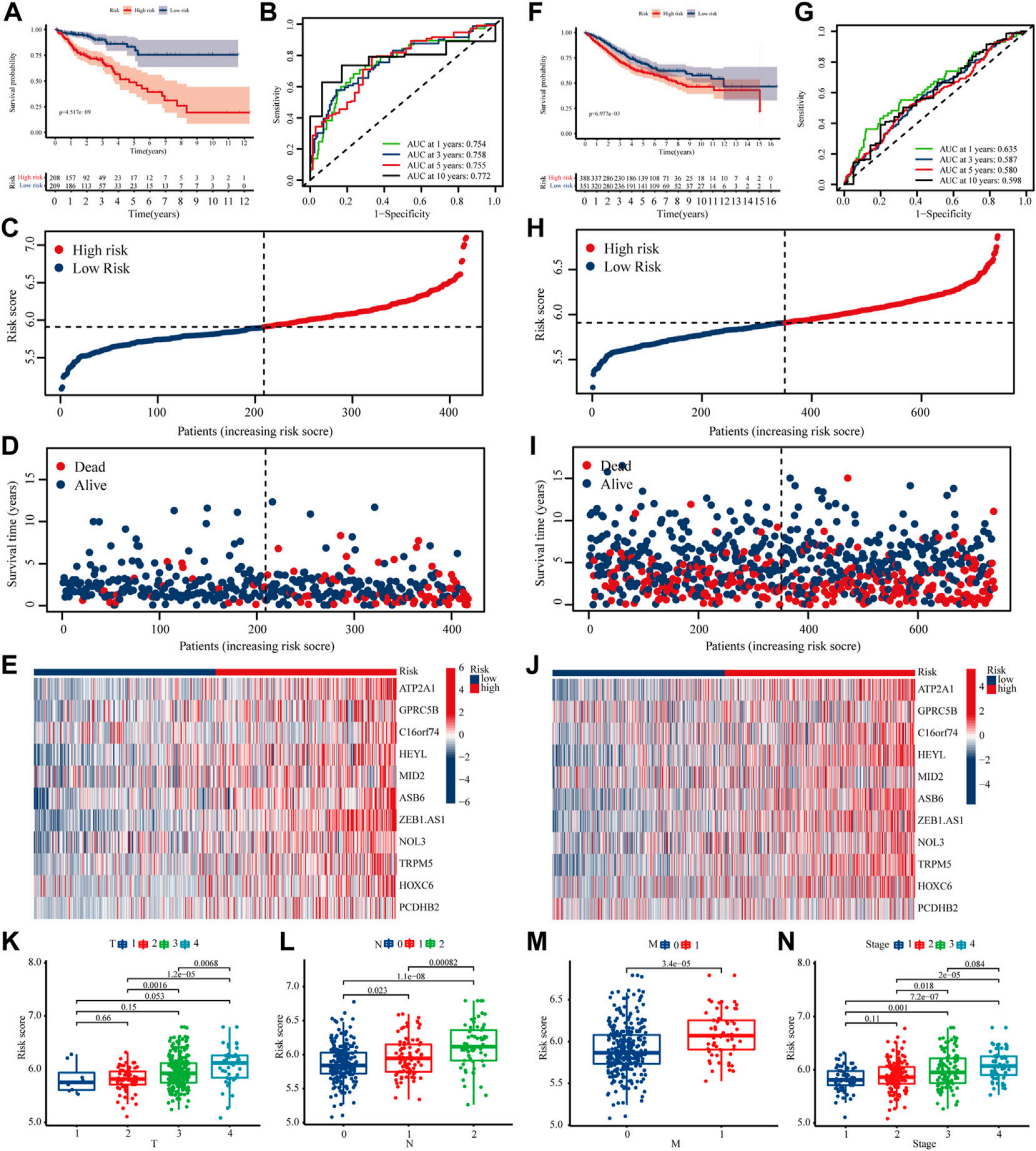
Validation of the risk model in the training set and testing set **(A,F)** K-M survival curves for high- and low-risk groups in training set and testing set **(B,G)** ROC curves for sensitivity and specificity of risk models used to predict 1-, 3-,5- and 10- year survival in training set and testing set **(C–D, H–I)** Scatter plot of m^7^G gene risk scores and patient survival, with red and blue dots representing high- and low-risk patients in training set and testing set **(E,J)** Expression of gene used to construct risk models in training set and testing set **(K–N)** Different TNM stage of colon patients in high- and low-risk scores.

The K-M survival curves showed that high-risk patients in train set had significantly lower survival rates than low-risk group (Figure 4A). The ROC curve demonstrated an AUC value of 0.754,0.758,0.755 and 0.772 for the survival probability of 1,3,5 and 10 years for colon patients. (Figure 4B). In addition, the number of patients who died increased significantly with increasing risk scoring (Figures 4C,D). Risk scoring also significantly differentiated patient prognosis in the external independent GEO dataset (Figures 4F–J)**.** Finally, our study suggested that high-risk colon cancer have relatively poorer TNM and Stage than patients in low-risk group (Figure 4K-N).

### ATP2A1 is Required for CRC Cell Survival

**FIGURE 5:**
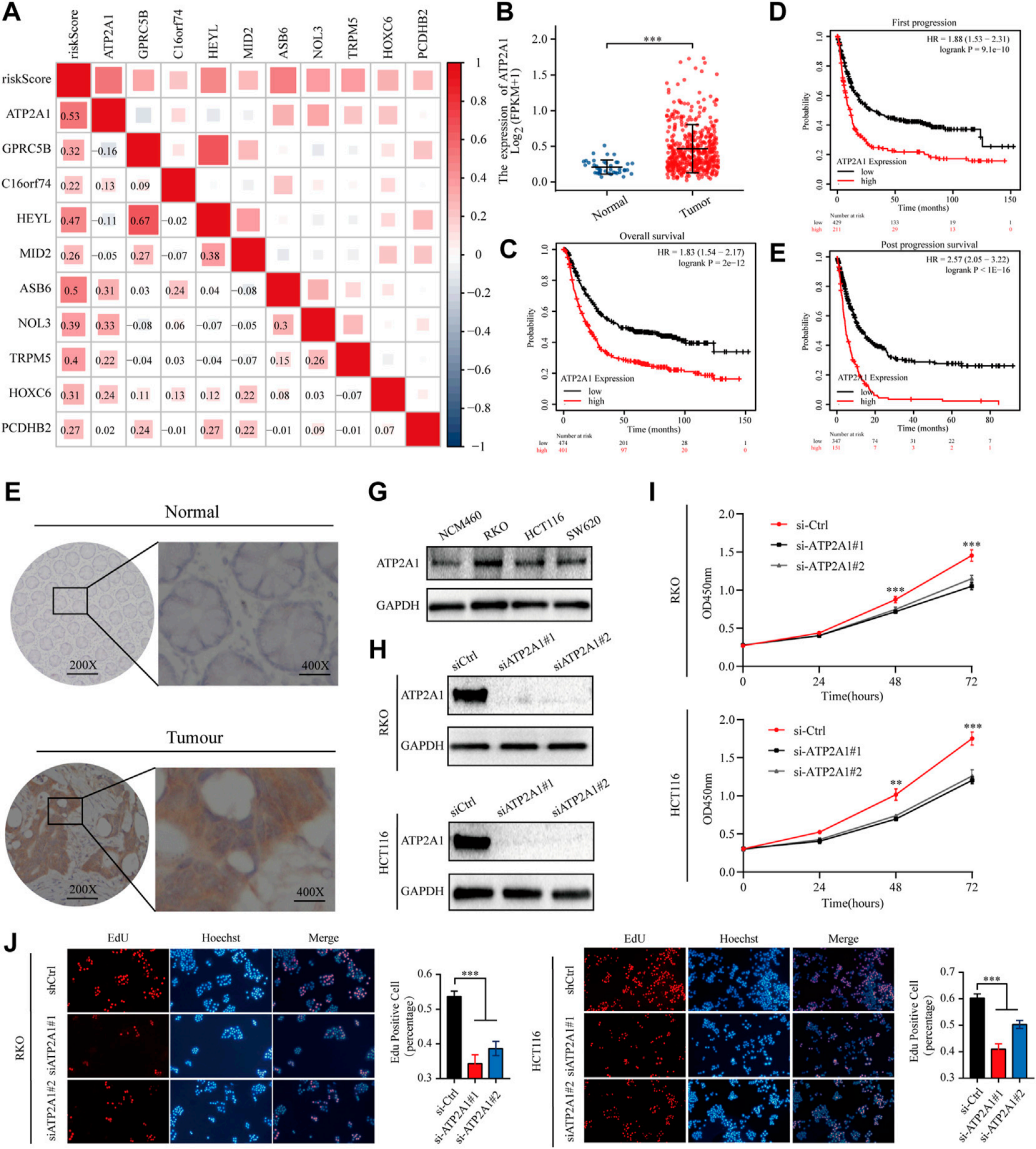
ATP2A1 is required for CRC cell survival **(A)** The relationship between ATP2A1 and risk model was showed in correlations matrices **(B)** The expression of ATP2A1 in CRC tissues and adjacent normal tissue of the TCGA database **(C–E)** Kaplan–Meier survival curves for CRC patients based on ATP2A1 mRNA expression **(F)**Representative images of immunostaining of ATP2A1 in primary CRC samples versus normal samples **(G)** Representative images of Western blot of ATP2A1 in RKO, HCT116, SW620 versus NCM460 **(H)** Western blot to show knockdown efficiency of ATP2A1 in RKO and HCT116 cells by two independent siRNAs **(I)**Cell proliferation of RKO cells or HCT116 cells **(J)** Edu assay to test the cell proliferation of control cells comparing to ATP2A1 knockdown cells.

Correlations matrices showed that ATP2A1 was most closely related to risk model (Figure 5A). Dates from the TCGA database showed that CRC tissues had higher expression levels of ATP2A1 than adjacent normal tissue (Figure 5B).K-M curves displayed that high expression of ATP2A1 is correlated with poor OS, FP and PPS of colon cancer patients (Figures 5C–E). Immunohistochemistry showed that ATP2A1 has higher expression in cancer than in adjacent normal tissue (Figure 5F). Western blot showed HCT116, RKO and SW620 have higher expression of ATP2A1 than NCM460 (Figure 5G). Two independent siRNAs (siRNA#1 and siRNA#2) were used to knockdown the expression of ATP2A1 in HCT116 and RKO (Figure 5H). CCK-8 and EdU assay result showed that the knockdown of ATP2A1 inhibit cell proliferation significantly (Figures 5I,J). These results illustrate that ATP2A1 is overexpressed in colon cancer, and may promote the progression of colon cancer.

### Prognostic Value of the Risk Model

**FIGURE 6:**
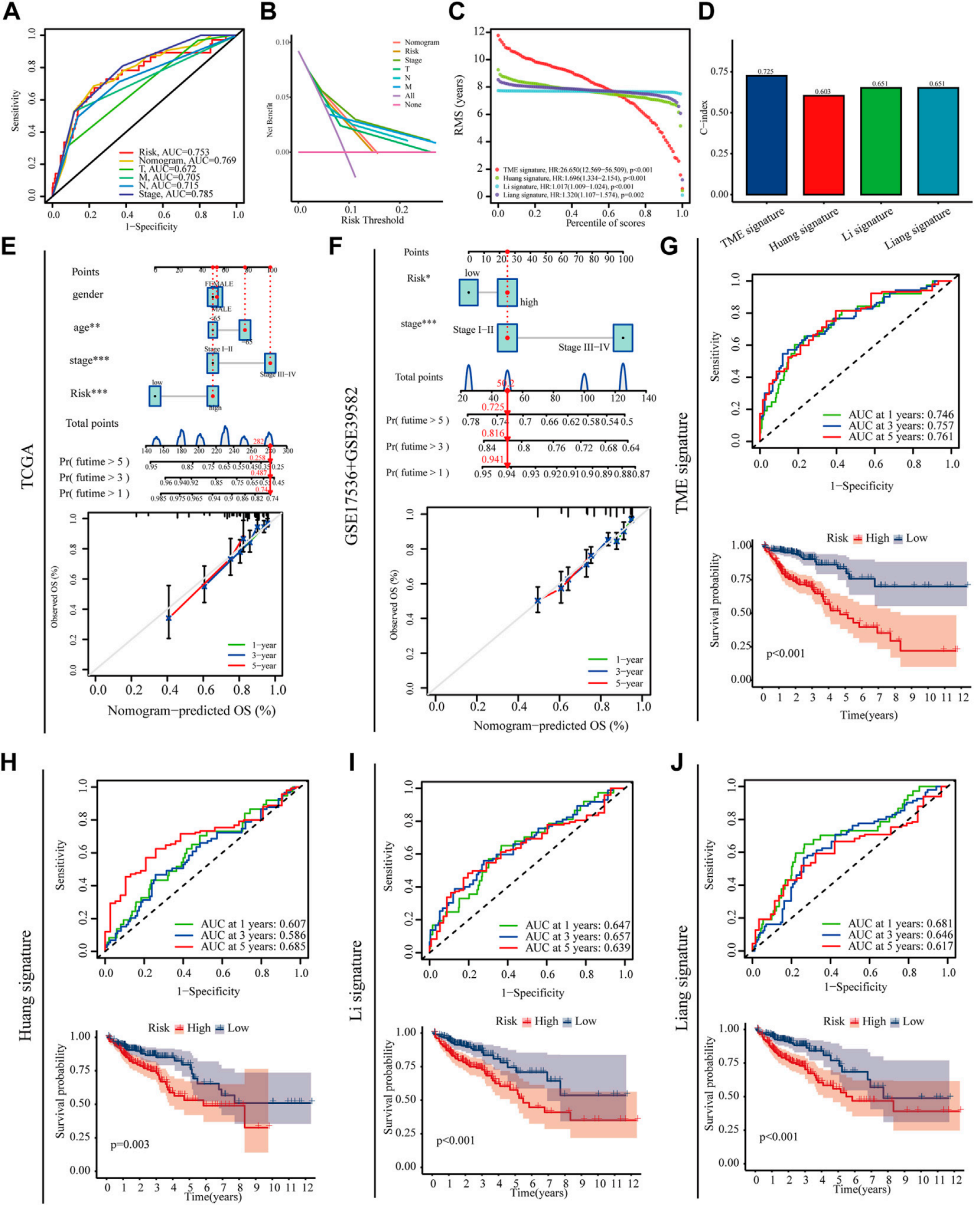
Prognostic value of the risk model. **(A,B)** Roc and DCA curves were performed to validate the risk score in predicting patient’ survival **(C,D)** RMS and C-index curves was used to compare the constructed model with existing models **(E,F)** Nomograms and calibration curves for predicting survival in colon cancer patients at 1, 3 and 5 years in training set and testing set **(G–J)** ROC and Kaplan–Meier survival curves was used to compare the constructed model with existing models.

This study then used a variety of methods to assess the predictive value of the prognostic model. High‐risk patients showed a poorer prognosis than low‐risk patients in both univariate Cox regression (hazard ratios [HR]: 16.008, 95% confidence interval [CI]: 7.955–32.212, *p* < 0.001) and multifactorial Cox regression (8.592 [CI]: 4.031–18.314, *p* < 0.001) (Supplementary Figure S2C, D)**.** PCA plots showed that the risk model is more capable than whole genome and the m^7^G genes to devised patients into high- and low-risk groups (Supplementary Figure S2E–G). The ROC and DCA curves indicate that the risk model has a higher accuracy and sensitivity in predicting patient prognosis compared to other clinical characteristics (Figures 6A,B). We further established prognostic nomograms including Stage, and risk score in both train set and test set to provide the survival probability of 1, 3, and 5 years for colon patients. Nomograms showed a good performance with a high AUC of 0.769, suggesting that it could be served as an effective tool for the prognostic evaluation of patients with CRC (Figure 6A). In addition, we constructed calibration curves, which showed that the predicted and actual survival rates were consistent with 1, 3, and 5 years (Figures 6E,F). We compared the model constructed in this study with existing models with ROC, C-index, K-M curves (Figure 6D,G-J). All results indicate that the constructed prediction model revealed a high efficacy for prognosis prediction.

### Immune Features in High- and Low-Risk Groups

**FIGURE 7:**
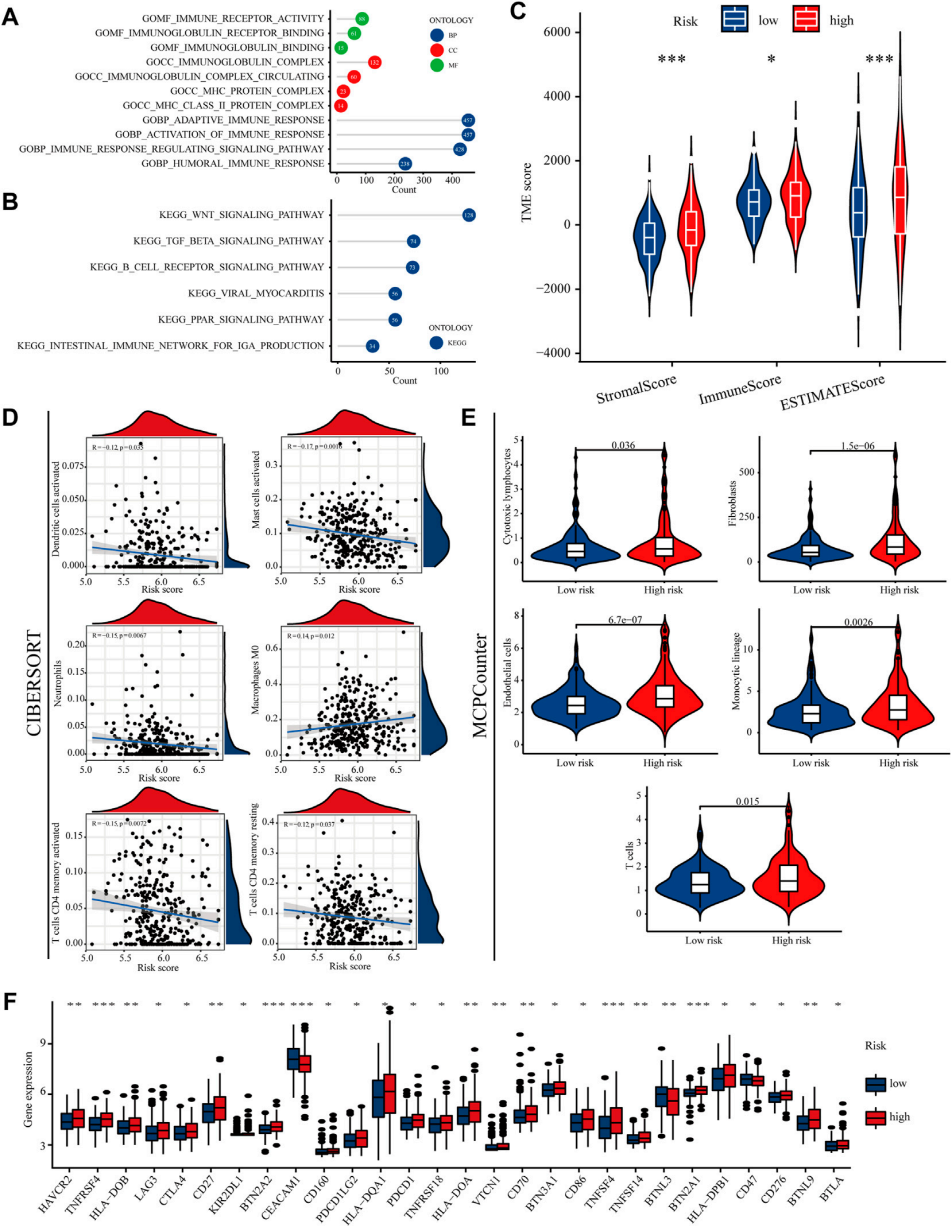
Immune features in high- and low-risk groups **(A,B)** Different GSEA result between high- and low-risk groups **(C)** immune microenvironment scores between the high- and low-risk groups **(D,E)** MCPcounter and CIBERSORT were used to calculate the relative proportions of immune cell subsets **(F)** The expression of immune checkpoints between the high- and low-risk groups.

GSEA showed that the immune response-related signature was prominently enriched in high-risk group (Figures 7A,B). Patients in high risk group had higher StromalScore、ImmuneScore and ESTIMATEScore than those in low risk group (Figure 7C). As shown in the diagrams, the risk score was positively correlated with M0 macrophages and negatively correlated with dendritic cells activated, mast cells activated, T cells CD4 + memory activated and neutrophils (Figures 7D,E). Immune checkpoint inhibitors are an important form of tumour immunotherapy (Hu et al., 2021). Most immune checkpoints have different expression between the high and low risk groups (Figure 7F). These results suggest that different risk score may have a direct impact on the efficacy of treatment, especially for immunotherapy.

### Mutations and Treatment Sensitivity Between High- and Low-Risk Groups

**FIGURE 8:**
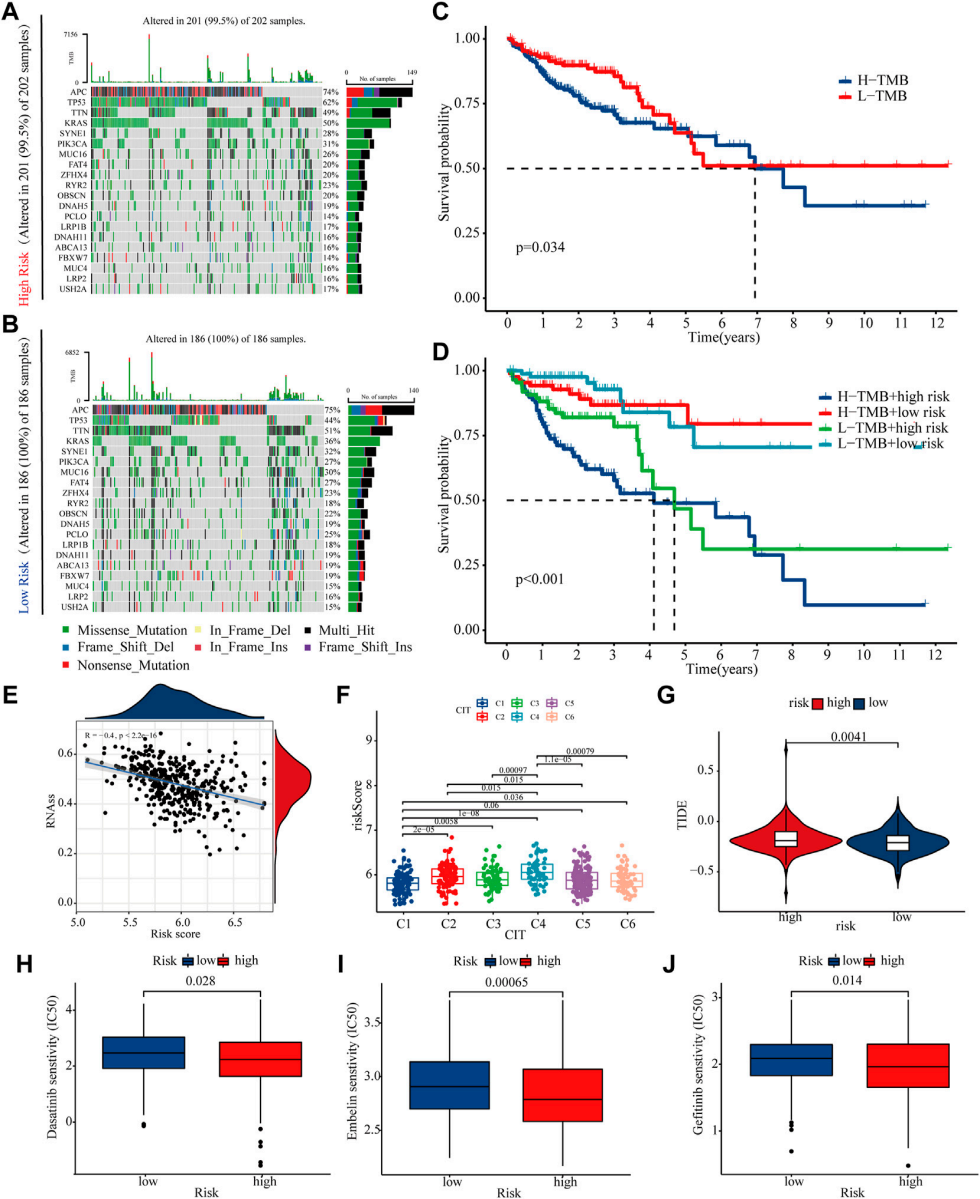
Mutations and treatment sensitivity between high- and low-risk **(A,B)** The top 20 genes’ Mutation rate in the high- and low-risk groups **(C,D)** K-M curves of risk score and tumour mutational load used to assess patient survival **(E)** The risk score is negatively correlated with the stem cell index **(F,G)** TIDE and GSE39582 database were used to validate patient sensitivity to immunotherapy between the high- and low-risk groups **(H–J)** The association between risk score and drug treatment sensitivity.

Because differences in gene mutations can dramatically affect treatment efficacies in colon cancer patients, so this study analyse the effect of high and low risk groups on mutation frequency. We listed the top 20 genes with highest mutation frequency, which showed that the mutation rate of KRAS, TP53 and PIK3CA in high-risk group was higher than low-risk group (Figures 8A,B). The K-M curve shows that patients with higher TMB have a worse prognosis, while high risk patients with high tumour mutation have the worst prognosis (Figures 8C,D). Tumour stem cells can associate malignant progression and immunotherapy tolerance in colon cancer. This study showed a negative correlation between risk score and stem cell index (R = -0.4, P < 2.2E-16), suggesting that high-risk patients have progressively lower stem cell characteristics and may have a better response to immunotherapy (Figure 7E). Combined with the results of immunotherapy typing in the GSE39582 dataset, there is a strong association between increased risk scores and sensitivity to different types of immunotherapies (Figure 8F). In addition, high risk patients have higher Tumour Immune Dysfunction and Rejection (TIDE) score, suggesting that high-risk patients may be less responsive to immunotherapy (Figure 8G). Finally, we found that patients in the high-risk group were significantly more sensitive to Gefitinib, Embelin and Dasatinib (Figures 8H–J). Taken together, these findings demonstrate that risk scores can accurately predict a patient’s sensitivity to multiple clinical agents.

## Discussion

Studies have shown that methylation plays an indispensable role in antitumour effects, and m^7^G is also a common mechanism of RNA methylation involved in the malignant biological behaviour of many tumours (Zhao et al., 2021a; Orellana et al., 2021). It has been reported that it also plays an important role in the antitumour immune process (Devarkar et al., 2016). DNA damage is strongly associated with metastasis in colon cancer and also predicts responsiveness to immunotherapy in colon cancer patients (Sun et al., 2019; Mauri et al., 2020). Previous studies have shown that methylation modulates the UV-induced DNA damage response to promote damage repair (Xiang et al., 2017). However, the effects of the m^7^G-related DDR genes on the immune microenvironment and immunotherapy in colon cancer have not been elucidated. Based on the TCGA database, an innovative prognostic model of m^7^G-related DDR genes was constructed to predict the prognosis and sensitivity to immunotherapy in colon cancer patients.

To validate the reliability of the model, the TCGA database was used as a training set and over 700 additional gastric cancer patients obtained from the GEO database were acted as a test set. The reliability of the model was further verified using ROC, C-index and DCA curves. Finally, the model constructed in this study also better than models constructed in other studies. All of these results indicate that the model constructed in this study is highly reliable in predicting patient prognosis.

We further analysed the mutation rates of ten risk genes in colon cancer patients and showed that ATP2A1 had the highest mutation rate. Thus, we suspect that ATP2A1 may be an important molecule influencing the malignant progression of colon cancer. Immunohistochemical staining and WB showed that the expression of ATP2A1 in cancer tissues was significantly higher than that in normal tissues adjacent to the cancer. The knockdown of ATP2A1 expression significantly inhibited the proliferation and migration of colon cancer cell lines. However, inconsistent with the findings of this study, the expression of ATP2A1 in breast cancer tissues appears to be lower than in normal tissues, which may be due to the tumour heterogeneity (Christodoulou et al., 2021).

Having established that the model had high predictive significance, the study went on to analysis model’s functions in guiding other clinical treatments of colon patients. Immunotherapy may improve prognosis in patients who have received inadequate conventional therapy, however, there is growing evidence that the tumour microenvironment has multiple immune escape mechanisms that impair antitumour immunity (Yang et al., 2020). Tumour-associated fibroblasts are closely associated with clinical treatment, and there is no consensus that they influence other cell populations in the tumour microenvironment, altering cancer progression, stromal remodelling, and drug resistance (Chen et al., 2021). M0 macrophages have been found to be significantly more abundant in tumour tissue than in adjacent normal tissue in patients with pancreatic ductal adenocarcinoma, and M0 macrophages are an independent predictor of poor prognosis (Xu et al., 2020). Mast cells have a dual effect on tumours, depending on the tumour type, cancer stage, the activation status of mast cells, etc. Increased mast cells are associated with longer patient survival in colorectal cancer (Mehdawi et al., 2016; Lichterman and Reddy, 2021). Dendritic cells are currently recognized as the most effective antigen-presenting cells, and patients have benefited from dendritic cell-based immunotherapy. National and international clinical trials evaluating dendritic cell-based tumour immunotherapy are currently underway (Steinman, 2012). This study revealed that the high-risk group had higher levels of M0 macrophages and fibroblasts, while activated mast cells, resting CD4^+^T cells and dendritic cells activated were less abundant, implying that the risk model constructed in this study can be used as a biomarker to predict the immune status of colon cancer.

Gene mutations are closely associated with tumour development and treatment sensitivity (Hanahan, 2022). In this study, we found that the frequency of TP53 and KRAS mutations was significantly higher in patients in the high-risk group than those in the low-risk group. KRAS mutations have been a significant impediment to colorectal therapy, with numerous targeted therapies failing to work due to secondary KRAS mutations (Zhao et al., 2021b). Even though various targeted inhibitors of KRAS have been subjected to extensive clinical trials in recent years, the number of patients who have benefited from them remains limited (Awad et al., 2021; Tanaka et al., 2021). Tumour immunotherapy is a new generation of tumour treatment after surgery, radiotherapy, and chemotherapy, and has benefited a large number of patients (Waldman et al., 2020). However, patients with KRAS mutations have a significantly worse prognosis following immunotherapy than patients with KRAS wild-type (Ricciuti et al., 2021). Drug sensitivity results showed that patients with a low-risk score were more sensitive to lapatinib and sorafenib. Additionally, numerous clinical trials have demonstrated that lapatinib and sorafenib improve patients’ prognoses (Bahrami et al., 2018; Wang and Fakih, 2021). In conclusion, the risk model constructed in this study serves as a guide for decision-making in targeted therapy and immune checkpoint inhibitor therapy for patients with colon cancer.

Despite the findings of this study, there are still some limitations. This study is mainly based on bioinformatics analysis and lacks experimental validation of the findings. Additionally, larger sample size is needed to verify the reliability of the model. In summary, a risk model for m7G-related genes was constructed in this study. This risk model can be used to predict the prognosis of colon cancer patients and to determine the efficacy of immunotherapy. These findings can provide a basis for prognosis prediction of colon cancer patients and to establish a reference value for colon cancer immunotherapy.

## Data Availability

The original contributions presented in the study are included in the article/Supplementary Material, further inquiries can be directed to the corresponding authors.
