# Supplementary figures and images for: m7G-Related DNA Damage Repair Genes are Potential Biomarkers for Predicting Prognosis and Immunotherapy Effectiveness in Colon Cancer Patients
Source: Front Genet. 2022 Jun 9;13:918159. doi: 10.3389/fgene.2022.918159 (PMC9218807; doi:10.3389/fgene.2022.918159)

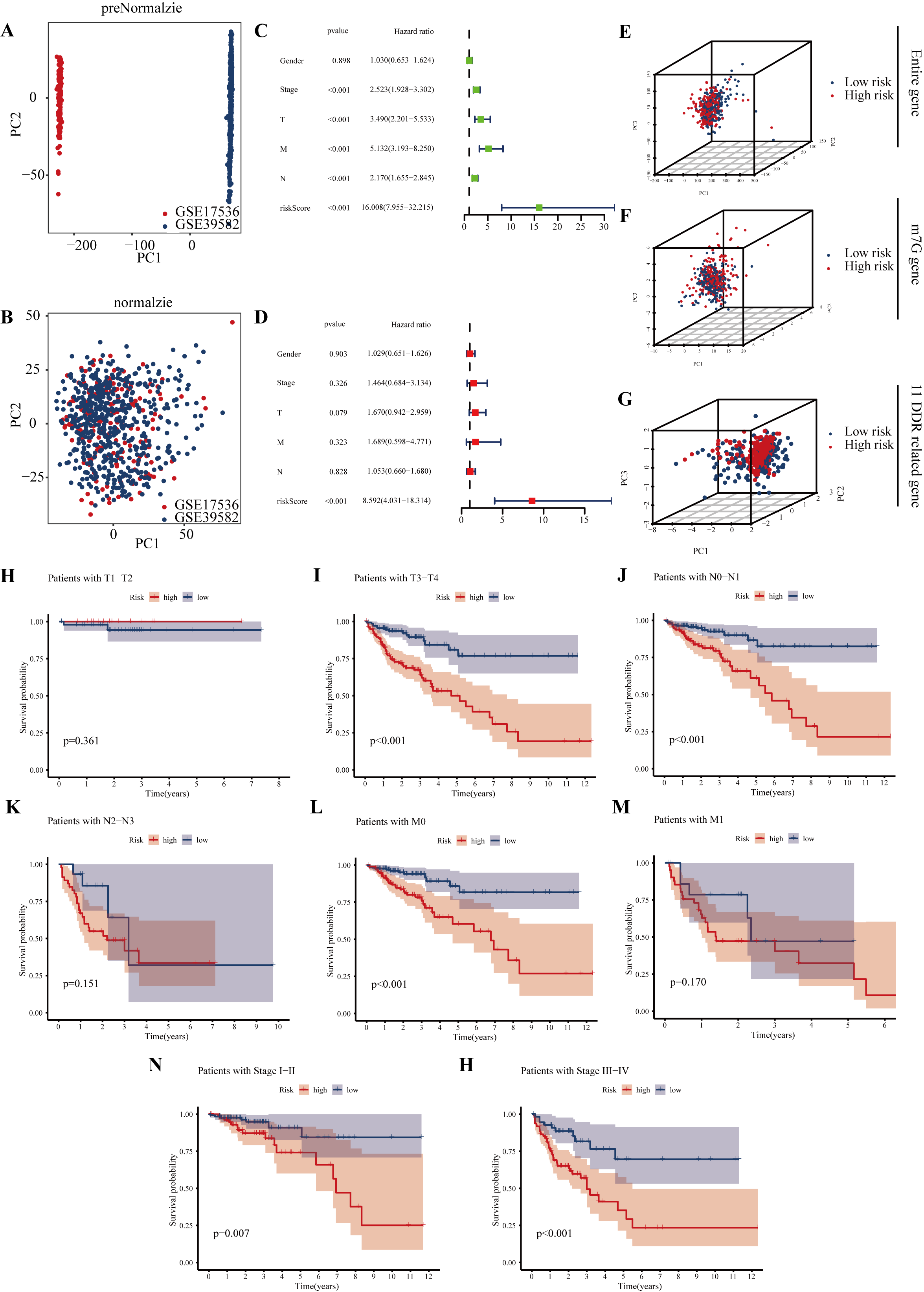

Supplement: Supplementary file 3 [file Image2.TIF]

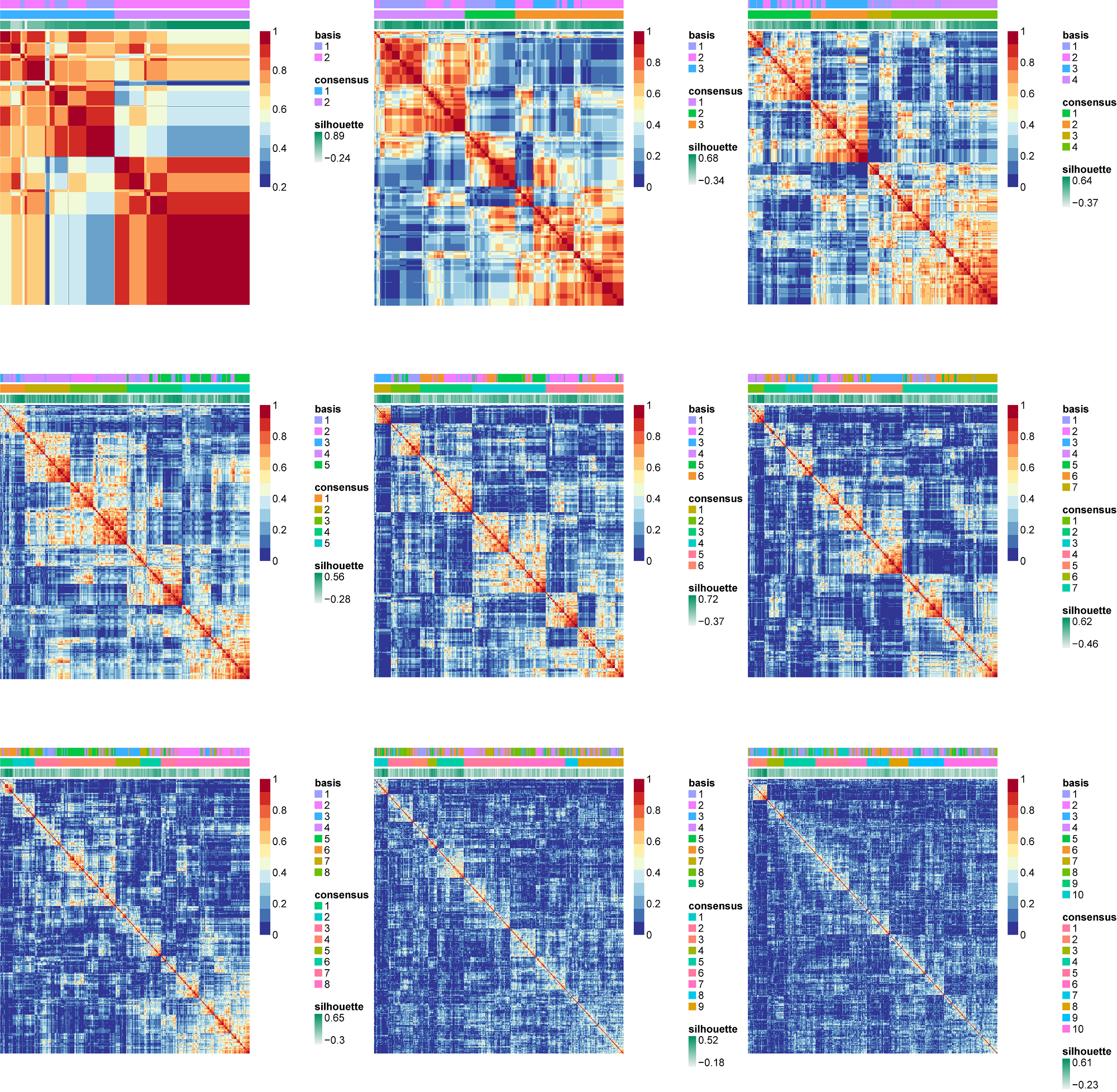

Supplement: Supplementary file 4 [file Image1.TIF]
